# Aquablation/AquaBeam Waterjet Therapy for Benign Prostatic Hyperplasia: Three‐Year Functional and Ejaculatory Outcomes in a Multicenter Real‐World Italian Cohort

**DOI:** 10.1111/luts.70088

**Published:** 2026-08-17

**Authors:** Angelo Porreca, Daniele D'Agostino, Davide De Marchi, Marco Giampaoli, Filippo Marino, Gian Maria Busetto, Antonio Amodeo, Paolo Corsi, Francesco Claps, Luca Di Gianfrancesco

**Affiliations:** ^1^ Department of Urology Humanitas Gavazzeni Bergamo Italy; ^2^ Humanitas University Rozzano Italy; ^3^ Department of Urology University of Foggia Foggia Italy; ^4^ Veneto Institute of Oncology ‐ IRCCS (IOV), Department of Oncological Urology Padua Italy

**Keywords:** Aquablation/AquaBeam, benign prostatic hyperplasia, ejaculatory preservation, lower urinary tract symptoms, waterjet ablation

## Abstract

**Objective:**

To evaluate the 3‐year functional outcomes of Aquablation/AquaBeam waterjet therapy for bladder outlet obstruction secondary to benign prostatic hyperplasia (BPH), with particular attention to urinary symptoms, quality of life, continence, ejaculatory function, and complications.

**Methods:**

We performed a retrospective analysis of a prospectively maintained multicenter database including 218 consecutive men who underwent Aquablation/AquaBeam for symptomatic BPH between January 2019 and January 2022 at three referral centers. Functional outcomes assessed preoperatively and during follow‐up included International Prostate Symptom Score (IPSS), IPSS quality‐of‐life item (IPSS‐QoL), maximum urinary flow rate (Qmax), post‐void residual urine (PVR), continence, and ejaculatory function. Median follow‐up was 36 months. Ejaculatory preservation was evaluated only in patients with preserved antegrade ejaculation at baseline.

**Results:**

Median age was 61 years (IQR 57–66), median prostate‐specific antigen (PSA) was 2.52 ng/mL (IQR 0.40–21.60), and median prostate volume was 55 mL (IQR 40–73). Median operative time was 56 min, while median catheterization time and length of hospital stay were both 48 h. Functional improvements were evident from the 3‐month follow‐up and remained stable through 36 months. At 36 months, median IPSS improved to 5 and median Qmax to 18 mL/s. Median PSA changed modestly from 2.52 ng/mL at baseline to 2.75 ng/mL at 36 months. No cases of de novo urinary incontinence were observed throughout follow‐up. Among patients with preserved antegrade ejaculation at baseline, 87% maintained antegrade ejaculation at 36 months. Most postoperative complications were minor (Clavien‐Dindo grade I‐II, 20.2%). One grade III rectal injury occurred (0.5%). Median hemoglobin decreased from 15.2 g/dL preoperatively to 14.0 g/dL before hospital discharge, and two patients required blood transfusion. During the 36‐month follow‐up, no surgical retreatment was required, whereas 11 patients (5.0%) received temporary medical retreatment.

**Conclusion:**

In this multicenter real‐world cohort, Aquablation/AquaBeam was safe and effective over 3 years, providing durable improvement in lower urinary tract symptoms and quality of life while preserving continence and antegrade ejaculation in most patients. These findings support Aquablation/AquaBeam as a valuable minimally invasive surgical option for selected men with BPH, particularly those who prioritize preservation of ejaculatory function. Longer‐term and comparative studies are warranted.

**Trial Registration:**

48281

## 1 Introduction

Benign prostatic hyperplasia (BPH) is a common condition in aging men, characterized by the non‐cancerous enlargement of the prostate gland, which often leads to bladder outlet obstruction (BOO) and lower urinary tract symptoms (LUTS). These symptoms have a significant negative impact on quality of life, affecting sleep, daily activities, and overall well‐being. Standard surgical treatment, such as holmium laser enucleation of the prostate (HoLEP), is effective in reducing LUTS but is often associated with loss of ejaculatory function [1].

In recent years, the Aquablation/AquaBeam technique, based on waterjet ablation technology, has emerged as a minimally invasive alternative for managing BOO caused by BPH. This technology uses a high‐velocity waterjet under real‐time imaging guidance to precisely ablate prostatic tissue, allowing for targeted removal with minimal damage to surrounding structures. This approach aims to offer relief from LUTS while preserving key aspects of sexual function, particularly ejaculation, which is a common concern for patients undergoing traditional BPH treatments [2, 3].

Although the evidence base for Aquablation/AquaBeam has expanded, the level of certainty required for the widespread implementation of novel surgical techniques remains a matter of ongoing discussion [4].

This study provides an analysis of the three‐year outcomes of Aquablation/AquaBeam waterjet therapy in a cohort of patients treated for BPH‐induced BOO. By evaluating changes in urinary symptoms, quality of life, and functional outcomes—including continence and ejaculatory function—this study aims to evaluate the safety, efficacy, and durability of Aquablation/AquaBeam over a medium‐term follow‐up period. These findings aim to provide further real‐world evidence on the role of Aquablation/AquaBeam in contemporary BPH management, particularly in patients prioritizing preservation of ejaculatory function.

## 2 Methods

### 2.1 Study Population

The study was based on a retrospective analysis of 218 consecutive patients with BOO due to BPH who were treated with Aquablation/AquaBeam waterjet ablation between January 2019 and January 2022. All procedures were conducted across three high‐volume referral centers specializing in BPH management. Patient data were derived from a prospectively maintained database to ensure consistency and accuracy in data collection.

### 2.2 Patients

Patient selection reflected routine clinical practice at the participating centres. Aquablation/AquaBeam was primarily offered to men with moderate‐to‐severe LUTS (IPSS ≥ 8) refractory to medical therapy who prioritized preservation of ejaculatory function. Patients presenting with absolute indications for BPH surgery, such as recurrent acute urinary retention, chronic indwelling catheterisation, or renal impairment secondary to bladder outlet obstruction, were not formally excluded but were more commonly managed with enucleative techniques according to centre practice and shared decision‐making. This selection strategy resulted in a low prevalence of preoperative urinary retention within the study cohort. This selection pattern is consistent with pivotal Aquablation trials (WATER and WATER II), in which patients with catheter‐dependent urinary retention were uncommon, as well as with real‐world registry data (ICARUS), highlighting that Aquablation/Aquabeam is frequently chosen for men with bothersome LUTS in whom preservation of sexual and ejaculatory function is a key treatment goal. Importantly, treatment allocation reflected shared decision‐making between patient and surgeon and adherence to contemporary guideline‐based practice, whereby the choice of surgical technique for BPH is individualized according to symptom burden, anatomical characteristics, comorbidities, and patient preferences—particularly regarding sexual and ejaculatory outcomes.

Patients receiving anticoagulant or antiplatelet therapy were not excluded; perioperative management was conducted according to institutional protocols, including temporary discontinuation or bridging when indicated.

A formal assessment of global sexual function was not performed. Preoperative ejaculatory status was documented, and analyses of ejaculatory preservation were limited to patients with preserved antegrade ejaculation at baseline.

### 2.3 Study Design

Multi‐centric, non‐controlled, observational, retrospective based on prospectively maintained database study. The study adheres to the STROBE recommendations for observational cohort studies.

### 2.4 Inclusion/Exclusion Criteria

Patients included in the study were adult men diagnosed with symptomatic BPH, confirmed through clinical evaluation, imaging, and laboratory tests. Criteria for inclusion were: presence of LUTS unresponsive to medical therapy, evidence of BOO based on clinical findings, complete follow‐up, signed informed consent. Exclusion criteria included prior prostate surgery, prostate cancer diagnosis, previous surgical intervention for BPH, severe medical comorbidities that would contraindicate the procedure.

In our cohort, patients with high PSA levels underwent appropriate diagnostic evaluation, including clinical assessment and imaging, and prostate biopsy when indicated to exclude malignancy. Only patients without evidence of prostate cancer were included in the analysis.

Patients were considered eligible for surgery in the presence of moderate‐to‐severe lower urinary tract symptoms (IPSS ≥ 8) that were refractory to medical therapy after an adequate treatment period. Additional indications included patient preference for surgical management, progression of symptoms despite pharmacological treatment, or the presence of complications related to bladder outlet obstruction.

### 2.5 Procedure

All Aquablation/Aquabeam waterjet ablation procedures were performed under general or spinal anesthesia, with patients positioned in lithotomy. The Aquablation/Aquabeam device was used to deliver high‐velocity waterjets, allowing precise and controlled ablation of the targeted prostatic tissue while sparing surrounding structures. This technique utilizes real‐time imaging and robotic assistance to minimize the risks of damage to adjacent tissues.

In all cases, at least one planned Aquablation/Aquabeam pass was performed; a second or additional pass was carried out at the discretion of the surgeon based on intraoperative assessment of residual adenoma and cavity adequacy.

Following waterjet ablation, the fragmented (“fluffy”) tissue was removed using a resection loop; hemostasis was achieved using bipolar coagulation [5], holmium laser hemostasis [6], or limited transurethral resection for hemostatic purposes, according to intraoperative findings and surgeon preference. The hemostatic technique used in each case was recorded and is reported in a dedicated table. Operative time, catheterisation duration, and length of hospital stay were systematically recorded as procedural variables.

### 2.6 Measurements

Functional outcomes were evaluated pre‐operatively and at multiple post‐operative time points (3 months, 1 year, and annually up to 3 years post‐surgery). The following measures were assessed: the IPSS, the IPSS‐Quality of Life (QoL) question; the maximum Urinary Flow Rate (Qmax); the Post‐Void Residual (PVR); the Continence status (evaluated based on patient‐reported continence levels and the need for any incontinence aids or medications).

A specific focus on sexual function was given to rates of ejaculation preservation, an important consideration in BPH treatment. Ejaculatory function was assessed preoperatively and at postoperative follow‐up visits at 3 months, 12 months, and annually up to 3 years. Ejaculatory preservation was defined as patient‐reported maintenance of antegrade ejaculation and was calculated exclusively in patients with preserved antegrade ejaculation at baseline. In addition, an exploratory analysis was performed to evaluate potential procedural factors associated with postoperative loss of antegrade ejaculation among patients with preserved ejaculation at baseline. Procedural variables analyzed included hemostatic technique and the number of Aquablation passes, using Fisher's exact test for categorical comparisons.

A formal assessment of erectile function using validated questionnaires (e.g., IIEF‐5) was not systematically performed in this cohort. However, clinical follow‐up records were reviewed for documentation of newly reported erectile dysfunction or initiation of pharmacological therapy for erectile dysfunction.

Complications following the Aquablation/Aquabeam waterjet ablation procedures were systematically documented and classified using the Clavien‐Dindo classification system. This classification facilitates objective and consistent reporting of post‐operative complications across clinical studies. Grade 1 Complications were defined as any deviation from the normal post‐operative course not requiring pharmacological treatment or surgical intervention. Grade 2 Complications require pharmacological treatment, such as antibiotics, analgesics, or anti‐inflammatory drugs, without necessitating invasive procedures. Grade 3 Complications involve those requiring surgical, endoscopic, or radiological intervention.

Histological specimens were obtained only in those cases where a limited transurethral resection or focal coagulation was performed for hemostasis after Aquablation/Aquabeam, allowing retrieval of sufficient tissue for pathological assessment. In the remaining patients, no representative tissue was available for histology.

Operative time was defined as the interval from insertion of the resectoscope into the urethra to removal of the instrument at the end of the procedure, including Aquablation/Aquabeam, tissue evacuation, and hemostasis.

### 2.7 Data Analysis

The sample size and the relative statistical power of the study were not calculated due to the nature of the study. Descriptive statistics were employed to summarize patient demographics and baseline characteristics. Continuous variables are reported as median (IQR) and categorical variables as *n* (%). Comparisons between preoperative and postoperative continuous variables were performed using paired statistical tests, selected according to data distribution. The paired Student's *t*‐test was used for normally distributed variables, while the Wilcoxon signed‐rank test was applied for non‐normally distributed data. Categorical variables were compared using the chi‐square test or Fisher's exact test, as appropriate. The distribution of continuous variables was assessed using the Shapiro–Wilk test. As continuous variables were not normally distributed, they are presented as median and interquartile range (IQR), whereas categorical variables are reported as frequencies and percentages. Statistical significance was set at *p* < 0.05.

### 2.8 Follow‐Up and Data Collection

All patients were followed for 36 months, with the primary endpoint assessed at 3 years postoperatively. Peri‐operative variables were recorded intraoperatively and during the immediate postoperative period until hospital discharge, including operative parameters and laboratory values such as hemoglobin level at discharge. Postoperative follow‐up was scheduled at 3, 6, 12, 24, and 36 months. At each visit, patients underwent clinical evaluation, symptom assessment using the International Prostate Symptom Score (IPSS), serum prostate‐specific antigen (PSA) measurement, and objective functional assessment including uroflowmetry and ultrasound examination. Postoperative complications were documented and graded according to the Clavien–Dindo classification, capturing both early (≤ 30 days) and late (> 30 days) adverse events.

### 2.9 Ethics Approval and Consent to Participate

This study was approved by the Local Ethical Committee (Comitato Etico Territoriale Lombardia 5, Via A. Manzoni 56, Rozzano 20 089) in the session of April 22nd 2025, no protocol (GAV 176/25).

All participants are required to provide written informed consent. Data collection will be conducted in accordance with the World Medical Association Declaration of Helsinki. The standard of care remains consistent with that provided according to international guidelines for patients participating in the study.

## 3 Results

### 3.1 Baseline Characteristics

A total of 218 consecutive patients with complete 36‐month follow‐up were included in the analysis after exclusion of three patients with incomplete data or loss to follow‐up. Median age was 61 years (IQR 57–66), median PSA was 2.52 ng/mL (IQR 0.4–21.6), and median prostate volume was 55 mL (IQR 40–73) (Table 1); the distribution of prostate volume is provided in Figure S1.

**TABLE 1:** Study population and baseline characteristics.

Variable	Value
*Study population*	
Patients treated with Aquablation/AquaBeam	221
Excluded due to incomplete data or loss to follow‐up, *n* (%)	3 (1.4)
Final cohort with complete 3‐year follow‐up, *n* (%)	218 (98.6)
*Baseline characteristics*	
Age, years, median (IQR)	61 (57–66)
PSA, ng/mL, median (IQR)	2.52 (0.40–21.60)
Prostate volume, mL, median (IQR)	55 (40–73)
Qmax, mL/s, median (IQR)	9.0 (6.2–11.0)
Middle lobe present, *n* (%)	92 (42.2)
Post‐void residual, mL, median (IQR)	90 (72.5–110.0)
Bladder lithiasis, *n* (%)	4 (1.8)
IPSS, median (IQR)	19 (16–21)
IPSS‐QoL, median (IQR)	4 (3–6)
Indwelling bladder catheter, *n* (%)	1 (0.5)
Urinary incontinence at baseline, *n* (%)	7 (3.2)
Urge urinary incontinence, *n*/*N* (%)	7/7 (100)
Stress urinary incontinence, *n*/*N* (%)	0/7 (0)
Pad use at baseline, *n* (%)	2 (0.9)
Hemoglobin, g/dL, median (IQR)	15.2 (14.4–15.7)
Receiving BPH‐related medication at baseline, *n* (%)	201 (92.2)
No BPH‐related medication at baseline, *n* (%)	17 (7.8)
Alpha‐blocker, *n* (%)	167 (76.6)
5‐alpha reductase inhibitor, *n* (%)	14 (6.4)
Combination therapy, *n* (%)	20 (9.2)

*Note:* Continuous variables are reported as median (IQR); categorical variables are reported as *n* (%). Elevated PSA values were investigated preoperatively with appropriate diagnostic work‐up, including prostate biopsy when indicated, and patients with confirmed prostate cancer were excluded.

Abbreviations: BPH, benign prostatic hyperplasia; IPSS, International Prostate Symptom Score; IQR, interquartile range; PSA, prostate‐specific antigen; Qmax, maximum urinary flow rate; QoL, quality of life.

### 3.2 Perioperative Outcomes

**FIGURE 1:**
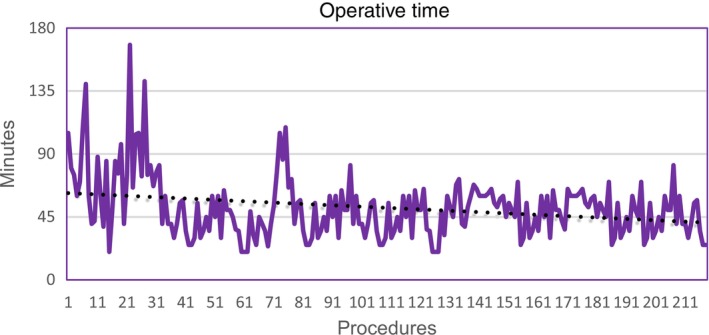
Operative time trendline.

Median operative time was 56 min (Figure 1), while median catheterization time and hospital stay were both 48 h (Table 2). Most patients were discharged within 2 days of surgery. Bipolar coagulation was the most frequently used hemostatic technique (56.4%), followed by holmium laser hemostasis (24.3%) and limited transurethral resection for hemostasis (19.3%) (Table 3).

**TABLE 2:** Peri‐operative and functional data.

Variable	Value
*Operative data*	
Operative time, min, median (IQR)	56 (31–168)
Catheterization time, h, median (IQR)	48 (24–480)
Length of hospital stay, h, median (range)	48 (24–600)
*Perioperative laboratory data*	
Hemoglobin, g/dL, median (IQR), preoperative	15.2 (14.4–15.7)
Hemoglobin, g/dL, median (IQR), postoperative^a^	14.0 (9.2–17.0)
Blood transfusion, *n* (%)	2 (0.9)
*PSA trend*	
PSA at baseline, ng/mL, median (IQR)	2.52 (0.40–21.60)
PSA at 3 months, ng/mL, median	2.36
PSA at 36 months, ng/mL, median	2.75
*Early complications (≤ 30 days)*	
Clavien–Dindo grade I, *n* (%)	18 (8.3)
Clavien–Dindo grade II, *n* (%)	26 (11.9)
Clavien–Dindo grade III, *n* (%)	1 (0.5)
— Rectal injury, *n* (%)	1 (0.5)
*Late complications (> 30 days)*	
Urethral stricture, *n* (%)	2 (0.9)
Bladder neck stenosis, *n* (%)	1 (0.5)
*Management of late complications*	
Treatment	Endoscopic cold urethrotomy/Holmium laser bladder neck incision
Outcome	Complete resolution without recurrence

*Note:* Continuous variables are reported as median (IQR) unless otherwise specified; categorical variables as *n* (%).

^a^ Postoperative hemoglobin measured before hospital discharge; Early complications defined as ≤ 30 days; late complications defined as > 30 days.

**TABLE 3:** Hemostatic techniques used following Aquablation/Aquabeam waterjet ablation.

Hemostatic technique	No. of patients, *n* (%)
Bipolar coagulation	123 (56.4)
Holmium laser hemostasis	53 (24.3)
Limited transurethral resection for hemostasis	42 (19.3)
Total	218 (100)

### 3.3 Functional Outcomes

**FIGURE 2:**
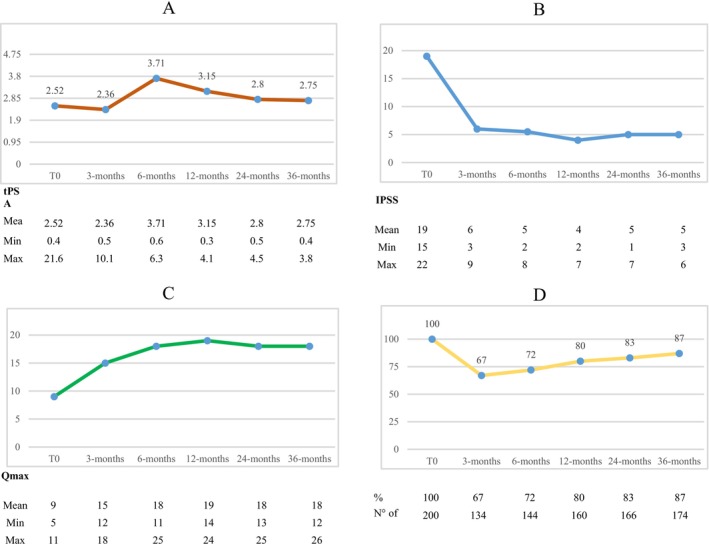
Functional outcomes after Aquablation during 36‐month follow‐up: A: TPSA, B: IPSS, C: Qmax, D: Antegrade ejaculation preservation.

Aquablation/AquaBeam resulted in rapid and durable functional improvement, with significant benefits evident from the 3‐month follow‐up and maintained throughout 36 months (Table 4) and (Figure 2).

**TABLE 4:** Functional outcomes over time after Aquablation.

Outcome	Baseline (T0)	3 months	6 months	12 months	24 months	36 months	*p* (T0 vs. 36 months)
IPSS	19 (IQR 16–21)	6 (IQR 4–8)	5 (IQR 3–8)	4 (IQR 2–6)	5 (IQR 3–7)	5 (IQR 4–6)	0.03
QoL (IPSS‐QoL)	4 (IQR 3–6)	2 (IQR 1–3)	1 (IQR 0–3)	1 (IQR 0–2)	1 (IQR 0–3)	1 (IQR 0–3)	0.03
Qmax (mL/s)	9 (IQR 6.2–11)	15 (IQR 13–17)	18 (IQR 14–20)	19 (IQR 15–22)	18 (IQR 15–22)	18 (IQR 15–23)	0.02
Post‐void residual (mL)	90 (IQR 50–110)	45 (IQR 25–60)	37 (IQR 30–48)	41 (IQR 20–55)	38 (IQR 25–60)	34 (IQR 20–45)	0.04
Total PSA (ng/mL)	2.52 (IQR 0.4–9)	2.36 (IQR 0.9–5.9)	3.71 (IQR 2.2–4.2)	3.15 (IQR 2.3–3.9)	2.80 (IQR 1.9–3.1)	2.75 (IQR 1.1–3.7)	0.28
Antegrade ejaculation	92%	67%	72%	80%	83%	87%	0.36

Median IPSS improved from baseline to 5 at 36 months, accompanied by sustained improvement in IPSS‐QoL. Similarly, median Qmax increased to 18 mL/s, while post‐void residual urine progressively decreased, indicating durable restoration of bladder emptying.

Median PSA decreased modestly at 3 months and remained stable thereafter, reaching 2.75 ng/mL at 36 months.

No cases of de novo urinary incontinence were observed during follow‐up, with continence preserved in all patients. Among patients with preserved antegrade ejaculation at baseline (92%), ejaculatory preservation reached 87% at 36 months following an initial postoperative decline and gradual recovery (over time).

Exploratory analyses found no significant association between postoperative ejaculatory function and hemostatic technique or the number of Aquablation passes (Table 5). Likewise, no cases of newly diagnosed erectile dysfunction requiring medical treatment were recorded.

**TABLE 5:** Exploratory analysis of procedural factors associated with ejaculatory outcomes (patients with antegrade ejaculation at baseline, *n* = 200).

Procedural variable	Ejaculation preserved (*n* = 174)	Ejaculation loss (*n* = 26)	*p*
Hemostatic technique (overall)			0.62
Bipolar coagulation	98 (56.3%)	14 (53.8%)	0.84
Holmium laser hemostasis	43 (24.7%)	5 (19.2%)	0.53
Limited TUR for hemostasis	33 (19.0%)	7 (26.9%)	0.36
≥ 2 Aquablation passes	74 (42.5%)	12 (46.2%)	0.74

### 3.4 Safety

Most postoperative complications were minor (Clavien–Dindo grade I–II, 20.2%) and resolved with conservative or standard medical management. One patient (0.5%) experienced a grade III rectal injury related to an unrecognized anatomical abnormality during transrectal ultrasound insertion; this was successfully managed surgically without long‐term sequelae. Median hemoglobin decreased modestly after surgery, and two patients required blood transfusion.

Late complications were uncommon, with urethral stricture occurring in two patients (0.9%) and bladder neck stenosis in one patient (0.5%). All were successfully treated endoscopically without recurrence during follow‐up.

### 3.5 Additional Findings

During the 36‐month follow‐up, two patients were diagnosed with ISUP grade group 1 prostate cancer and were managed with active surveillance. No oncological progression or surgical retreatment was observed. Temporary medical retreatment for LUTS was required in 11 patients (5.0%).

## 4 Discussion

This study reinforces the growing body of evidence supporting Aquablation/Aquabeam waterjet ablation as a minimally invasive and effective option for the management of LUTS due to BPH. Our three‐year follow‐up data confirm substantial and durable improvements in functional outcomes, together with a favorable safety profile, underscoring Aquablation/Aquabeam as a strong alternative to traditional resective therapies, which are often associated with higher rates of ejaculatory and sexual dysfunction. These findings are consistent with contemporary narrative reviews and comparative overviews of emerging BPH interventions, in which Aquablation/Aquabeam is highlighted as a key technology within the modern surgical armamentarium alongside HoLEP and Rezūm [3, 7, 8, 9].

The improvements in functional outcomes observed in our cohort, particularly the reduction in IPSS and the increase in Qmax, are in line with the main Aquablation/Aquabeam studies, including WATER and WATER II [10, 11], and the individual‐patient meta‐analysis by Elterman et al. [12]. In our series, the median IPSS at 36‐month follow‐up was significantly reduced, with a median score of 5 (IQR 3–21), mirroring the magnitude of symptom relief reported in pivotal trials and long‐term extensions, where IPSS reductions of approximately 15–17 points and sustained benefits over time have been documented [13, 14]. Similarly, WATER II demonstrated significant improvements at 12 months, with a mean IPSS decrease of 17 points, a Qmax increase of 12.5 mL/s and a PVR reduction of 171 mL in patients with elevated baseline PVR; at 5 years, only 3% of patients required surgical retreatment [11]. In contrast, no patient in our cohort required retreatment during the 3‐year follow‐up, further supporting the medium‐term durability of Aquablation/Aquabeam in routine clinical practice.

More recently, a pooled analysis of the WATER, WATER II, OPEN WATER, FRANCAIS WATER and JAPAN PMS studies confirmed sustained improvements in IPSS, quality of life, Qmax and PVR and reinforced the overall safety and effectiveness of Aquablation/Aquabeam across diverse populations and prostate anatomies [15]. These multicountry data complement our findings, placing our real‐world Italian experience within a broader international framework.

In the pivotal double‐blind, multicentre RCT comparing Aquablation/Aquabeam with transurethral resection of the prostate (TURP), reductions in IPSS were similar between the two groups, with no significant differences in IPSS, Qmax or PVR at 6 months. Urodynamic sub‐studies of the WATER trial at 6 months showed marked reductions in pdetQmax (35 and 34 cm H_2_O, respectively) and significant improvements in BOO index in both arms [16]. Our results confirm that such improvements in IPSS and Qmax remain sustained at 3 years in a real‐world setting, indicating robust symptom relief across a range of prostate sizes. Moreover, Aquablation/Aquabeam has consistently shown shorter resection times compared with TURP (e.g., 4 vs. 27 min), which may contribute to reduced operative trauma and faster recovery [10]. A recent prospective comparative study including Aquablation/Aquabeam, TURP and HoLEP further supports this functional advantage, reporting the greatest IPSS improvement in the Aquablation/Aquabeam group, despite larger baseline prostate volumes, with preserved erectile function and significantly better maintenance of ejaculation compared with both TURP and HoLEP [17].

One of the most compelling strengths of Aquablation/Aquabeam in our series is the high rate of ejaculatory function preservation, a priority outcome for many men selecting surgical treatment for BPH. At 3 years, 87% of our patients maintained antegrade ejaculation, closely paralleling the 80%–90% preservation rates reported in WATER and WATER II [10, 11], and in more recent pooled and prospective comparative analyses [12, 15, 17]. In contrast, TURP is well known to carry a high incidence of ejaculatory dysfunction, with the pivotal RCT reporting anejaculation rates of 36% for TURP compared with only 10% for Aquablation/Aquabeam [10]. This difference is echoed in systematic and narrative reviews focusing on ejaculatory function preservation across minimally invasive surgical treatments for BPH, where Aquablation/Aquabeam consistently emerges as one of the techniques with the most favorable ejaculatory profile [3, 7]. Mechanistically, this benefit is attributable to the precise, image‐guided waterjet ablation of adenomatous tissue with preservation of critical ejaculatory and neurovascular structures. Preservation of ejaculatory function after Aquablation/Aquabeam is primarily related to avoidance of injury to the ejaculatory ducts and periurethral tissues near the verumontanum; disruption of these structures has been shown to increase the risk of postoperative anejaculation [18, 19]. Although often better described in the context of erectile function, adjacent neurovascular tissue conveys autonomic innervation that supports ejaculatory physiology and underscores the value of precise, image‐guided contour planning to minimize collateral injury [20, 21]. In order to explore potential determinants of ejaculatory outcomes, we performed an exploratory analysis evaluating the association between postoperative loss of antegrade ejaculation and selected intraoperative variables, including hemostatic technique and the number of Aquablation passes. No statistically significant correlation was identified between ejaculatory dysfunction and the hemostatic strategy used (bipolar coagulation, holmium laser hemostasis, or limited transurethral resection), nor with the number of treatment passes performed. These findings suggest that, within the technical variations adopted in our cohort, ejaculatory preservation after Aquablation appears to be largely independent of the hemostatic modality used. However, given the relatively small number of patients experiencing ejaculatory dysfunction, this exploratory analysis should be interpreted cautiously. Larger prospective studies specifically designed to investigate anatomical and procedural predictors of ejaculatory outcomes are warranted to better clarify the determinants of ejaculation preservation after Aquablation.

Sexual function after surgical treatment for BPH includes both erectile and ejaculatory components, which may be differentially affected depending on the surgical technique used. In the present study, our primary focus was the preservation of antegrade ejaculation, as this outcome represents one of the principal functional advantages associated with Aquablation and is frequently a key determinant in patient preference when selecting a surgical treatment for BPH. In our cohort, ejaculatory preservation remained high throughout follow‐up, reaching 87% at 3 years, confirming the favorable profile of Aquablation in maintaining this aspect of sexual function. A formal evaluation of erectile function using validated instruments such as the International Index of Erectile Function (IIEF) was not systematically performed in this study. However, a review of postoperative follow‐up records did not reveal new clinically reported erectile dysfunction requiring pharmacological treatment during the follow‐up period. Although this observation may indirectly suggest that erectile function was largely preserved, the absence of standardized erectile function questionnaires limits the ability to draw definitive conclusions regarding postoperative erectile outcomes. Consequently, the present analysis of sexual function should be interpreted as primarily focused on ejaculatory preservation rather than global sexual function. Future prospective studies incorporating validated measures of erectile function, such as the IIEF‐5, together with detailed assessment of ejaculatory parameters, will be important to provide a more comprehensive evaluation of sexual outcomes after Aquablation and to further clarify its impact on overall sexual health in men undergoing surgical treatment for BPH.

The safety profile observed in our study is also consistent with the existing literature, including the WATER trials, the individual‐patient meta‐analysis, and more recent pooled and real‐world series [8, 10, 11, 12, 15, 22]. Most of the complications in our cohort were minor (Clavien–Dindo Grade 1–2) and transient, with only 0.5% Grade 3 events and no long‐term sequelae. The WATER trial reported a lower rate of persistent adverse events in the Aquablation/Aquabeam group compared with TURP at 3 months, again supporting the minimally invasive nature of the procedure [10]. In WATER II, bleeding‐related events occurred in 13.9% of patients, but transfusion rates remained relatively low; our results are consistent with this profile, with a low transfusion requirement despite a clinically significant hemoglobin drop similar to that reported in a systematic review of hemoglobin changes after Aquablation/Aquabeam [12, 23].

Recent data have further refined our understanding of perioperative bleeding and hemostatic strategies in Aquablation/Aquabeam. A dedicated study on tranexamic acid (TXA) showed that limited TUR with focal cautery provides effective hemostasis after Aquablation/Aquabeam, whereas routine TXA use had only minimal impact on hematuria and was not associated with thromboembolic events, suggesting that TXA should not be used systematically when limited TUR is performed [24]. In parallel, a contemporary safety‐focused series from Mainez Rodríguez et al. highlighted that, despite its robotic precision, Aquablation/Aquabeam is not entirely free from serious complications (including rare events such as rectal injury) and therefore requires an appropriate learning curve and strict adherence to technical recommendations [25]. Our multicentre experience, characterized by low serious complication rates, is in keeping with these observations and underlines the importance of adequate training and centre expertise.

From a clinical perspective, understanding the nature and mechanisms of early and late complications associated with Aquablation/Aquabeam is essential for appropriate patient counseling and procedural planning. Bleeding‐related events remain among the most commonly reported early complications after Aquablation/Aquabeam and are primarily attributable to the non‐thermal, mechanical nature of tissue ablation, which does not intrinsically provide coagulation. In our cohort, bleeding complications and transfusion rates were low and comparable to those reported in pivotal trials and real‐world series, and were effectively managed with focal transurethral coagulation or laser hemostasis without long‐term sequelae. This profile is consistent with existing evidence indicating that, despite a measurable hemoglobin drop, clinically significant bleeding after Aquablation/Aquabeam is generally transient and manageable in experienced centres.

Rectal injury represents a rare but potentially serious complication of Aquablation/Aquabeam and is mechanistically linked to the proximity of the posterior prostatic capsule to the rectal wall during image‐guided waterjet ablation. In our series, rectal injury was an exceptional event, reflecting strict adherence to real‐time ultrasound guidance, appropriate treatment depth planning, and growing operator experience. The very low incidence observed is in line with contemporary reports and underscores the importance of the learning curve and meticulous procedural execution.

Late complications such as urethral stricture and bladder neck contracture were uncommon in our cohort. Notably, their incidence appears lower than that historically reported for thermal‐based resective techniques, such as TURP or HoLEP, where thermal injury and subsequent fibrosis play a central pathogenic role. The largely non‐thermal nature of Aquablation/Aquabeam may contribute to reduced cicatricial remodeling of the urethra and bladder neck, potentially explaining the favorable stricture and contracture rates observed in our study and others.

Overall, while Aquablation/Aquabeam is not free from complications, our data confirm that significant early and late adverse events are infrequent, manageable, and compare favorably with conventional resective approaches, particularly with respect to long‐term obstructive sequelae.

The question of Aquablation/Aquabeam safety in more fragile or elderly populations has been specifically addressed by the ICARUS real‐world database, which confirmed that Aquablation/Aquabeam is feasible and effective even in men aged ≥ 80 years, with acceptable morbidity and functional outcomes [22]. Although our cohort was younger (median age 61 years), these external data broaden the generalisability of Aquablation/Aquabeam, indicating that the technique can be safely offered to selected elderly patients within an integrated management strategy for BPH [9].

Our procedural metrics, including a median operative time of 56 min and short catheterisation and hospitalization durations (median 48 h), are also in line with results from WATER, WATER II and more recent multicentre experiences, where short operative times and brief stays highlight the efficiency and minimally invasive character of Aquablation/Aquabeam [8, 10, 11, 12, 15, 17]. These characteristics, combined with durable functional improvements and sexual function preservation, position Aquablation/Aquabeam as an attractive option within guideline‐driven care pathways. Recent national and expert consensus guidelines, such as those from the French Male LUTS Committee, now include Aquablation/Aquabeam among the recommended surgical treatments for BOO related to BPH, particularly in men prioritizing ejaculatory function [26].

Our findings and those from long‐term and pooled datasets collectively suggest that Aquablation/Aquabeam provides durable improvements in IPSS and Qmax over at least 3–5 years. In the WATER and WATER II trials, IPSS reductions remained stable over 5 years, while Qmax improvements of approximately 125% were observed with Aquablation/Aquabeam versus 89% with TURP [14]. Although the overall retreatment rate was slightly higher for Aquablation/Aquabeam (5.1% vs. 1.5% for TURP), the rate in large prostates (WATER II cohort) was as low as 3% at 5 years [11, 14, 15]. In this context, our absence of retreatment at 3 years is reassuring and suggests that Aquablation/Aquabeam may be particularly valuable in anatomically challenging cases.

Procedural refinements also appear to influence outcomes. An analysis combining data from WATER I and WATER II showed that two or more Aquablation/Aquabeam passes, compared with a single pass, resulted in IPSS scores that were approximately four points lower and Qmax values 5 mL/s higher at 24–36 months, without compromising ejaculatory function [27]. In our practice, we routinely performed a second or additional treatment pass guided by real‐time assessment of the residual adenoma in order to optimize cavity creation. This strategy likely contributed to the favorable functional results observed in our cohort and is consistent with the concept that protocolised multi‐pass approaches can enhance voiding outcomes without increasing morbidity.

Finally, Aquablation/Aquabeam waterjet ablation did not produce a marked PSA decline in our series, which is biologically plausible given the nature and location of the treated tissue. Aquablation/Aquabeam selectively targets adenomatous tissue in the transitional zone responsible for BOO, while largely sparing the peripheral and central zones where much of the PSA‐producing glandular tissue resides. As a result, overall PSA‐producing tissue volume is relatively preserved. The precision of the robotically guided waterjet limits collateral damage compared with less selective resection or vaporization techniques, and PSA, being secreted predominantly by epithelial cells in the peripheral zone, may therefore remain relatively stable after Aquablation/Aquabeam. Unlike certain laser therapies or more extensive TURP, Aquablation/Aquabeam does not typically vaporize large amounts of PSA‐contributing tissue. In summary, the limited PSA decline observed after Aquablation/Aquabeam reflects its targeted, tissue‐sparing mechanism, which prioritizes functional improvement and anatomical preservation over maximal volume reduction.

Taken together, our medium‐term data, when viewed alongside recent narrative reviews, pooled analyses, guideline recommendations and real‐world registries [7, 8, 9, 12, 15, 22, 26], support Aquablation/Aquabeam as a mature, evidence‐based option within contemporary BPH surgery, particularly for men in whom preservation of ejaculation and global sexual function is a primary treatment goal [3, 7, 8, 9, 18, 19].

## 5 Limitations

This study, while providing valuable insights into the medium‐term efficacy and safety of Aquablation/Aquabeam waterjet ablation for treating BOO in patients with BPH, has several limitations that should be acknowledged.

Firstly, this was a retrospective analysis, which inherently introduces potential biases related to patient selection and data collection. Although data were prospectively maintained, the absence of randomization limits the ability to control for confounding factors that might affect outcomes, such as baseline patient characteristics or variations in technique across centers.

Secondly, the follow‐up period was limited to a median of 3 years. While this timeframe allows for meaningful assessment of medium‐term outcomes, it does not provide information on the long‐term durability of Aquablation/Aquabeam waterjet ablation. Consequently, further studies with extended follow‐up periods are necessary to determine whether the functional improvements and high ejaculation preservation rates observed in this study are sustained over time.

Additionally, this study did not include a control group of patients undergoing alternative treatments for BPH, such as transurethral resection of the prostate (TURP) or laser‐based procedures, limiting direct comparisons. A comparative study design would help clarify the relative advantages and disadvantages of Aquablation/Aquabeam waterjet ablation against established treatment options, particularly regarding outcomes like symptom relief, complication rates, and preservation of sexual function.

Despite the high preservation rate indicates that patients maintained relatively stable ejaculatory function following the Aquablation/Aquabeam procedure, the specific quantitative MSHQ‐EjD scores were not provided in the study summary. Further studies with detailed MSHQ‐EjD scores could provide additional insights into the specific aspects of ejaculatory function preserved with Aquablation/Aquabeam over a longer‐term follow‐up.

Despite the study reported low rates of complications and successful resolution of all adverse events, the sample size may still be insufficient to capture rarer but potentially significant complications or adverse effects. Larger multicenter studies or meta‐analyses would provide a more comprehensive safety profile of the procedure.

Postoperative PSA kinetics were not correlated with long‐term retreatment risk in this study, and the limited PSA reduction observed after Aquablation/Aquabeam cannot be interpreted as a predictor of late durability due to the relatively short follow‐up period.

Although these aspects are discussed in the literature, tranexamic acid use, frailty assessment, and age‐specific outcome analyses were not part of the study design and therefore represent limitations of the present analysis.

Lastly, a formal assessment of erectile function using validated questionnaires was not performed; therefore, conclusions regarding overall sexual function should be limited to ejaculatory outcomes only.

In summary, while the results of this study are promising, its retrospective nature, lack of long‐term data, absence of a comparative group, and potential underrepresentation of rare complications indicate that further randomized, controlled studies with extended follow‐up are warranted to fully establish the clinical value and durability of Aquablation/Aquabeam waterjet ablation in the management of BPH.

## 6 Conclusions

Aquablation/Aquabeam waterjet ablation demonstrated favorable safety and efficacy for the treatment of BOO secondary to BPH in this cohort. The procedure resulted in significant and durable improvements in LUTS over a three‐year follow‐up period, while maintaining high rates of urinary continence and ejaculatory function preservation. The overall complication profile was low, with most adverse events being mild and resolving without long‐term sequelae.

These findings support Aquablation/Aquabeam as a minimally invasive surgical option capable of providing sustained symptom relief while preserving important functional outcomes. However, longer‐term follow‐up and prospective comparative studies are needed to further clarify its role within the surgical management of BPH.

## Author Contributions

Conceptualization: Angelo Porreca, Luca Di Gianfrancesco, Daniele D'Agostino. Methodology: Filippo Marino, Davide De Marchi. Validation: Filippo Marino; formal analysis: Filippo Marino. Investigation: Luca Di Gianfrancesco. Resources: Antonio Amodeo, Francesco Claps, Paolo Corsi. Data curation: Luca Di Gianfrancesco. Writing – original draft preparation: Luca Di Gianfrancesco, Filippo Marino. Writing – review and editing: Daniele D'Agostino, Gian Maria Busetto. Visualization: Davide De Marchi, Marco Giampaoli, Antonio Amodeo, Francesco Claps. Supervision: Angelo Porreca. All authors have read, reviewed and agreed to the published version of the manuscript.

## Funding

This study was supported by “Ricerca Corrente” who provided funding from the Italian Ministry of Health to cover publication costs.

## Ethics Statement

This study was approved by the Local Ethical Committee (Comitato Etico Territoriale Lombardia 5, Via A. Manzoni 56, Rozzano 20 089) in the session of April 22nd 2025, no protocol (GAV 176/25). All participants are required to provide written informed consent. Data collection will be conducted in accordance with the World Medical Association Declaration of Helsinki. The standard of care remains consistent with that provided according to international guidelines for patients participating in the study.

## Consent

As this study does not involve identifiable images or personal/clinical details that could compromise participant anonymity, the “Consent for Publication” is not applicable.

## Conflicts of Interest

The authors declare no conflicts of interest.

## Supporting information


**Figure S1:** Distribution of baseline prostate volume in the study cohort.

## Data Availability

The data that support the findings of this study are available from the corresponding author upon reasonable request.
