# Supplementary material for: Aquablation/AquaBeam Waterjet Therapy for Benign Prostatic Hyperplasia: Three‐Year Functional and Ejaculatory Outcomes in a Multicenter Real‐World Italian Cohort
Source: Low Urin Tract Symptoms. 2026 Aug 17;18(5):e70088. doi: 10.1111/luts.70088 (PMC13481998; doi:10.1111/luts.70088)
Supplement: Supplementary file 1 [file LUTS-18-e70088-s001.docx]

Supplementary Figure S1. Distribution of baseline prostate volume in the study cohort
